# Ramsay-Hunt syndrome and subsequent sensory neuropathy as potential immune-related adverse events of nivolumab: a case report

**DOI:** 10.1186/s12885-019-6444-0

**Published:** 2019-12-16

**Authors:** Takashi Sakoh, Mami Kanzaki, Atsushi Miyamoto, Sayaka Mochizuki, Toshiyuki Kakumoto, Kenichiro Sato, Yoshikazu Uesaka, Kazuma Kishi

**Affiliations:** 1 Department of Respiratory Medicine, Toranomon Hospital (Branch), 1-3-1 Kajigaya, Takatsu-ku, Kawasaki-shi, Kanagawa 213-8587 Japan; 2 Department of Neurology, Toranomon Hospital (Branch), 1-3-1 Kajigaya, Takatsu-ku, Kawasaki-shi, Kanagawa 213-8587 Japan; 3 Okinaka Memorial Institute for Medical Research, 2-2-2 Toranomon Minato-ku, Tokyo, 105-8470 Japan

**Keywords:** Immune checkpoint inhibitor, Intravenous immunoglobulin, Lung cancer, Neurological adverse events, Nivolumab, Ramsay-Hunt syndrome, Sensory neuropathy

## Abstract

**Background:**

Nivolumab is an immune checkpoint inhibitor (ICI) and is used for the treatment of advanced non-small cell lung cancer (NSCLC). Several immune-mediated neurological adverse events associated with ICIs have been reported to date, such as Guillain-Barré syndrome. Nivolumab-associated neurological adverse events can vary, and their etiology remains unclear.

**Case presentation:**

A 72-year-old man with NSCLC was treated with nivolumab as a second-line therapy. After 13 rounds of nivolumab therapy, he presented with Ramsay-Hunt syndrome (RHS) followed by acute ataxic sensory neuropathy. Antiviral therapy for Varicella-Zoster virus and prednisolone resulted in partial improvement of RHS, while almost no recovery was observed in the sensory neuropathy. However, the sensory ataxia significantly improved after intravenous immunoglobulin (IVIg) therapy, and interestingly, the facial palsy associated with RHS also improved. The neurological manifestations, nerve conduction study result, and imaging findings supported that dorsal root ganglia were the primary lesion site of acute ataxic sensory neuropathy.

**Conclusions:**

Our case presented with the comorbidity of RHS and subsequent ataxic sensory neuropathy after nivolumab therapy to whom IVIg was effective. Our case suggested the wide variability of possible neurological symptoms, and the potential usefulness of IVIg to sensory ataxic neuropathy, seen in cancer patients with ICI treatment.

## Background

Nivolumab is an immune checkpoint inhibitor (ICI) that targets programmed cell death-1 (PD-1) receptors, and is used for the treatment of advanced non-small cell lung cancer (NSCLC) in patients who did not respond to first-line chemotherapy [1, 2].

Several neurological adverse events associated with ICIs have been reported, such as neuropathy, encephalitis, chronic inflammatory demyelinating polyneuropathy, Guillain-Barré syndrome (GBS), myasthenia gravis, etc. [3–7], the underlying mechanisms of which are not yet fully understood. It is suspected that the loss of T cell inhibition via PD-1 blockade leads to impaired self-tolerance due to prior subclinical autoimmune disease or cross-reactivity of nervous system antigens with those of tumors [8], and this is considered to result in immune-mediated neurological adverse events [6]. In addition, since the use of ICIs can result in infections including opportunistic meningitis or Varicella-Zoster virus (VZV) reactivation [7, 9], ICI-associated neurological disorders can also be mediated by infectious etiologies. Thus, the spectrum of nivolumab-associated neurological adverse events could be wide. However, due to the lack of accumulation of the literature of clinical detailed and appropriate findings in the real world, the detailed etiologies of the neurological adverse events by ICIs are a sparsely investigated topic.

Here, we report the case of a 72-year-old man with NSCLC, who presented with Ramsay-Hunt syndrome (RHS) and acute sensory neuropathy, both of which may be associated with the use of nivolumab.

## Case presentation

**Fig. 1:**
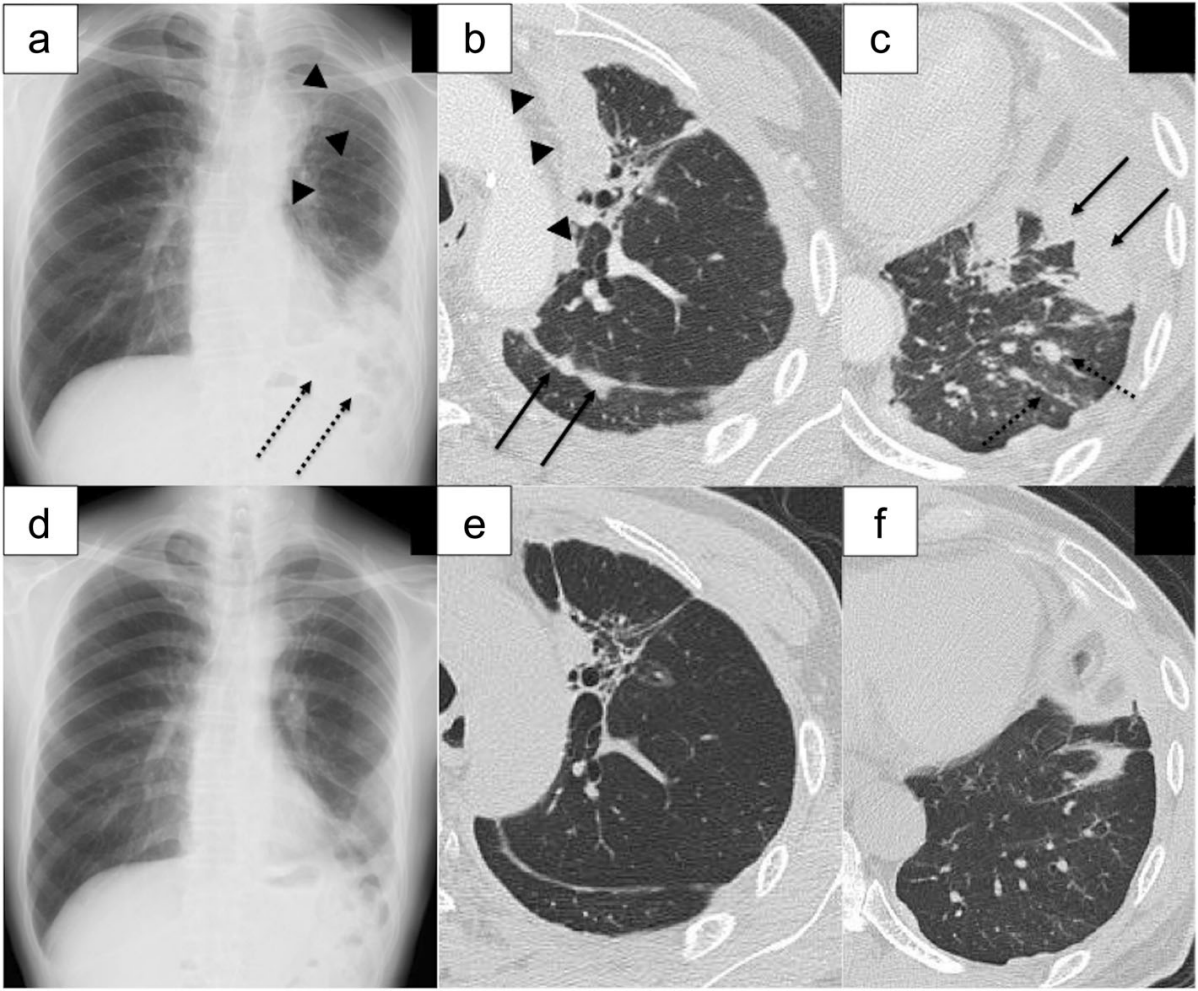
Chest imaging findings, Chest imaging at baseline (**a**-**c**), and after 13 rounds of nivolumab treatment (**d**-**f**), On the chest x-ray, primary tumor was shown in the upper lung field in contact with upper mediastinum (arrow heads), and disseminated tumor masses were mainly identified in the left lower lung field as a consolidated area (black dotted heads) (**a**) and were improved after nivolumab therapy (**d**). On the chest computed tomography image, the primary lesion in the left upper lobe adjacent to mediastinum (black arrow heads), disseminated multiple masses in the thoracic cavity (black solid arrows), and pleural and interlobular septal thickening due to lymphatic spread of tumors (black dotted arrows) (**b**, **c**) were all improved after nivolumab therapy (**e**, **f**)

A 71-year-old man with severe cough presented with left-sided pleural effusion. After thoracentesis, he was diagnosed with lung adenocarcinoma with malignant effusion without activating epidermal growth factor receptor mutations and anaplastic lymphoma kinase rearrangements (clinical T1aN3M1a, stage IVa). He was a former smoker with a smoking index of 15 pack-years. He was an electrical engineer with a history of occupational X-ray exposure. Four cycles of carboplatin (area under the blood concentration-time curve of 6 mg/mL・min) and pemetrexed (PEM, 500 mg/m^2^) were administered, followed by talc pleurodesis. Thereafter, six cycles of maintenance therapy with PEM were performed. Disease progression after 9 months from his first chemotherapy session led him to receive nivolumab as a second line therapy (Fig. 1a-c). He received nivolumab (3 mg/kg) every 2 weeks for a total of 13 rounds. Nivolumab resulted in a partial response only with grade 3 lymphocytopenia (approximately 300–400 cells/μL) (Fig. 1d and f).

Four days after the 13th nivolumab administration, he developed otitis externa on his left ear and it got worse. Moreover, an additional 4 days later, he developed unsteadiness on standing with acute onset. His initial neurological findings revealed sensory ataxia of his four extremities: positive Romberg’s test, decreased vibration sense of the bilateral ankles, and poor proprioception of his bilateral upper limbs, with no significant limb weakness, pyramidal signs, or decrease in superficial sensation. Four days after the onset of unsteadiness, his temperature increased (39.0 °C) without headache or meningism. Brain magnetic resonance imaging (MRI) was not suggestive of carcinomatous meningitis or metastatic lesions. Contrast-enhanced MRI was performed to monitor the spinal cord and brachial plexus, and yielded no abnormal findings.

**Fig. 2:**
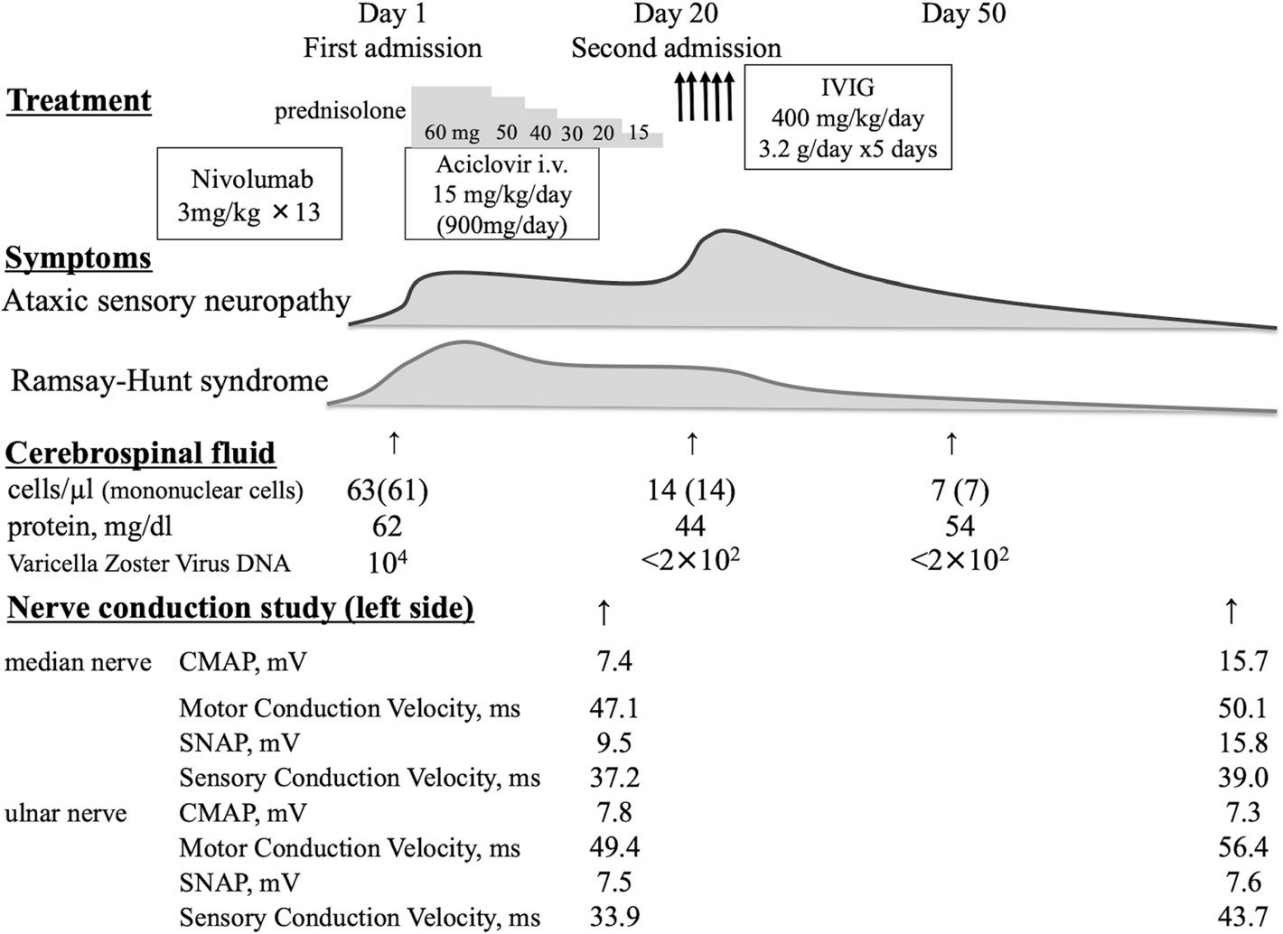
Clinical time course after developing Ramsay-Hunt syndrome and subsequent sensory neuropathy, Abbreviations: IVIg: intravenous immunoglobulin, CMAP: compound motor action potential amplitude, SNAP: sensory nerve action potential amplitude

His otitis externa was diagnosed as VZV infection of the left external auditory canal due to VZV reactivation. He clearly declared his childhood experience of chicken pox and no recent close contact with patients who suffered from VZV infection, and his serum immunoglobulin (Ig) status against VZV at his initial admission soon after developing his symptoms showed a significantly elevated level of serum IgG (44.2 antibody index by the enzyme immunoassay [EIA]: normal range of < 2.0) and a normal level of serum IgM (0.16 antibody index by the EIA: normal range of < 0.80). He was admitted to our hospital 9 days after his initial symptom presentation and started aciclovir (15 mg/kg/day: 900 mg/day) intravenously (day 1) for 7 days (Fig. 2). Cerebrospinal fluid (CSF) assessment on day 1 revealed an elevated white blood cell count (63 cells/mm^3^), protein level (62 mg/dl), and IgG index score (0.76): this is simply calculated by the following equation:

$$ IgG\  index=\left(\frac{CNS\  IgG}{CNS\  Alb}\right)\bullet \left(\frac{serum\  Alb}{serum\  IgG}\right) $$

to see whether there may be intrathecal increase of IgG, and its normal range is 0.34–0.58 [10]. Meanwhile, no malignant cells were found and the glucose level was normal (63 mg/dl). In addition, the level of VZV-DNA was significantly increased (10,000 copies/ml, normal range < 200 copies/ml). Following the development of otitis externa, left-sided facial nerve palsy was observed from day 2 and worsened to the most severe level (House-Brackmann grade 5) [11] on day 3. These clinical features and laboratory data corresponded to RHS. We additionally administered 60 mg of prednisolone on day 3.

Nerve conduction studies (NCS) were performed on day 14 to evaluate his sensory ataxia. These studies revealed a significant reduction in the sensory nerve conduction velocity (SCV) with fewer sensory nerve action potentials (SNAPs) on the left median (SCV 37.2 m/s, SNAP 9.5 μV) and ulnar nerves (SCV 33.9 m/s, SNAP 7.5 μV), and no significant abnormal findings in compound motor action potentials. Sensory-dominant ataxic neuropathy was considered to be the most likely etiology. While the sensory ataxia was not improved by acyclovir and prednisolone treatments, a partial response was observed in the facial nerve palsy due to RHS, maintaining a House-Brackmann grade of 5. We also performed somatosensory evoked potential (SEP) studies to assess neurological function, which revealed neuropathy instead of myelopathy. We opted to reduce the prednisolone dose and discharge the patient under close observation on day 16.

Since his unsteadiness worsened even after completion of aciclovir and reduction of prednisolone, he was re-admitted to our hospital on day 20 (Fig. 2). We re-evaluated the CSF on day 22 and found slight improvements in the white blood cell count (14 cells/mm^3^) and protein level (44 mg/dl), and VZV-DNA was not detected. Spinal MRI again revealed no significant changes to support VZV encephalopathy or myelitis. Notably, 12 antibodies of paraneoplastic neurological syndromes (PNS) (Hu, Yo, Ri, PNMA2, CV2, Amphiphysin, anti-Tr/DNER, GAD65, ZIc4, Titin, SOX1, and Recoverin; examined using an immunoblotting method by BML Inc.) and anti-ganglioside antibodies including anti-ganglioside complexes in the serum were all negative on day 22. Since the sensory ataxia persisted even after treatment with aciclovir and a short course of prednisolone, we suspected an immune-mediated etiology other than VZV infection. Thus, we performed one course of intravenous immunoglobulin (IVIg) therapy (400 mg/kg/day for 5 days [days 23–27]). Within 2 days, his facial nerve palsy had significantly improved (House-Brackmann grade 2), and his deep sensation also began to recover, which enabled him to take Romberg’s and Mann’s postures. Eventually, he was able to stand on his left leg at 15 days after the administration of IVIg (day 38). We confirmed abnormal NCS features with the delayed SCV and decreased SNAP amplitudes recovered on the median nerve (day 91).

During the treatment for RHS and ataxic sensory neuropathy, the status of lung cancer remained stable without the use of any chemotherapeutic agents.

## Discussion and conclusion

While nivolumab provides us with a novel strategy to treat several cancers, acute-onset neurological adverse events including peripheral neuropathy, GBS, meningitis, neuromuscular disorders, and encephalitis have also been reported to present in 0.93% of 3763 advanced melanoma patients receiving nivolumab with or without ipilimumab [5]. The present case developed both RHS and sensory-dominant ataxic neuropathy after receiving nivolumab as a second-line therapy. The combination of RHS and ataxic sensory neuropathy and the significant improvements in the related symptoms by IVIg as observed in our case have not been documented previously.

RHS, which typically manifests as a triad of ipsilateral facial paralysis as a neurological deficit, ear pain, and vesicles in the auditory canal or on the auricle [12, 13], is often induced by reactivation of prior VZV infection in the geniculate ganglion [14], and has not been documented previously as an adverse event of ICIs. With regard to the pharmacological effect of nivolumab on the immune system, infectious adverse events should be inhibited in patients treated with ICI, as nivolumab induces an antitumor response by activating T cells [6]. This seems to be contradictory since the VZV reactivation requires disrupted T-cell mediated VZV immunity [15]. However, in the real world setting, previous reports have indicated that serious infection developed in 7.3% of 740 melanoma patients who received ipilimumab, nivolumab, or pembrolizumab. Although being rare, disseminated or facial VZV infection was indeed observed in patients who received ICIs [9] with unknown etiology. Mild lymphocytopenia seen in our case might assist VZV reactivation [16]. Hence, there is no wonder to suspect the RHS due to VZV reactivation as one of the possible adverse events of nivolumab.

A previous study reported a case of sensory neuropathy as an adverse reaction of ICIs [7], but the detailed clinical presentation remains unknown. None of the previous studies reported the mechanism of sensory-dominant ataxic neuropathy induced by ICIs. Physical, electrophysiological, and imaging findings comprehensively revealed that the cervical dorsal funiculus or dorsal root ganglia (DRG) were the exact disordered and causative legions of neuropathy, because no cervical lesions were found in MRI studies and because the SEP and NCS studies indicated the evidence of neuropathy, but not myelopathy. One possible explanation for nivolumab-induced sensory ataxia is the enhanced cytotoxicity of CD8-positive lymphocytes against self-antigens within DRG, which may occur due to cross-reactivation by nivolumab and be enhanced by VZV reactivation. It was reported in some earlier studies that hantavirus-infected cells strongly express programmed cell death-ligand 1 (PD-L1) and PD-L2 to prevent damage by immune cells, and moreover, that lymphocytic choriomeningitis virus-infected cells were damaged by activated CD8-positive cells via blockade of PD-1 and PD-L1 binding [17]. These observational studies may indicate the importance of the PD-1 cascade in viral immunity, namely, that the loss of T cell inhibition via the cascade could attack DRG via self-antigens that cross-reactivate with other antigens.

Our case showed significant responsiveness to IVIg treatment. IVIg treatment is reported to be effective for treating neuropathies due to nivolumab use [6, 7], and may also be effective in treating specific cases of VZV-related neurological disorders [18, 19], although there are no sufficient data on the efficacy of IVIg for the treatment of VZV infection. Overt improvement in persistent facial paralysis with IVIg treatment may provide evidence that cytotoxic etiology, following VZV reactivation, is involved in its pathogenesis [18].

Taken together, the possible hypothesis for our case comorbid with sensory ataxia and VZV infection simultaneously is suspected as follows: Firstly, VZV in the patient’s geniculate ganglion was reactivated by nivolumab; secondly, VZV was inhibited by aciclovir and prednisolone but the facial nerve dysfunction remained; thirdly, the DRG were damaged by CD8-positive cells activated by nivolumab due to cross-reactivity of antigens within the DRG, leading to subsequent sensory ataxic neuropathy; and finally, these immune-related neurological insufficiencies recovered after IVIg treatment. Although PNS is also known as a disease that mainly targets the DRG [20] and it remains a differential diagnosis for our patient, we believe that PNS is unlikely because the lung adenocarcinoma was well controlled and the tests for the 12 antibodies of PNS were negative.

Our report has several limitations. First, we cannot strictly determine that the sensory ataxic neuropathy in our case was due to the direct adverse event associated with nivolumab, or due to the subsequent comorbidity of VZV infection. Second, while we discussed the VZV involvement mainly as the reactivation in a close association with the nivolumab use and suggested the explicable hypothesis, its association was not strictly proven in our case. Third, because of his single time of the IgG and IgM status at-index admission, the reactivation of VZV due to other concomitant events, or the possibility of VZV primoinfection, were not strictly ruled out in our patient, based on his concurrent coexistence of lung cancer and the past history of chemotherapies prior to nivolumab.

In conclusion, our case presented with RHS and sensory dominant ataxic neuropathy following nivolumab administration, suggesting the wide variability of neurological symptoms potentially observed under ICI usage. Our case confirmed the significant efficacy of IVIg in the treatment of the partially persisting facial nerve palsy and sensory ataxia, suggesting the potentially considerable use of IVIg as a therapeutic option in the treatment of similar neurological symptoms.

## Data Availability

Data sharing is not applicable for this case report, as no datasets were generated during the current study, which was based on clinical observations.

## Abbreviations

CSF: Cerebrospinal fluid
DRG: Dorsal root ganglia
EIA: Enzyme immunoassay
GBS: Guillain-Barré syndrome
ICI: Immune checkpoint inhibitor
Ig: Immunoglobulin
IVIg: Intravenous immunoglobulin
MRI: Magnetic resonance imaging
NCS: Nerve conduction studies
NSCLC: Non-small cell lung cancer
PD-1: Programmed cell death-1
PD-L1: Programmed cell death-ligand 1
PEM: Pemetrexed
PNS: Paraneoplastic neurological syndrome
RHS: Ramsay-Hunt syndrome
SCV: Sensory nerve conduction velocity
SEP: Somatosensory evoked potentials
SNAP: Sensory nerve action potential
VZV: Varicella-Zoster virus
